# Herbal Medicine in Primary Healthcare in Germany: The Patient's Perspective

**DOI:** 10.1155/2012/294638

**Published:** 2012-12-31

**Authors:** Stefanie Joos, Katharina Glassen, Berthold Musselmann

**Affiliations:** ^1^Department of General Practice and Health Services Research, University Hospital Heidelberg, Voßstraße 2, 69115 Heidelberg, Germany; ^2^Family Medicine Practice, Academic Teaching Practice, University of Heidelberg, Hauptstraße 120, 69168 Wiesloch, Germany

## Abstract

Herbal medicine (HM) is one of the most widely used Complementary and Alternative Medicine (CAM) therapies throughout the world. The WHO has recognized HM as an essential component of primary healthcare. The aim of this study was to explore patients' attitudes towards using HM, their sources of information and the role of costs. Within a qualitative research approach, semi-standardized interviews with 18 patients using HM were conducted and analyzed according to Mayring's content analysis. Patients highlighted their active role and perceived autonomy choosing HM. Most interviewees experienced HM as better, with more sustainable effects and fewer side effects compared to conventional medicine. All media, family, friends, and healthcare professionals were reported as sources of information. Some patients complained that doctors and pharmacists have insufficient knowledge of HM. Most patients expressed their regret that HM is not reimbursed by statutory health insurances but also their general willingness to pay extra for HM. The main challenge for German primary care, besides the reintroduction of reimbursement, is the promotion of knowledge and skill development in HM. This is to ensure patient safety and work in partnership with patients. Appropriate strategies for education must be tailored to the specific needs of health professional groups.

## 1. Introduction 

Herbal medicine (HM) is one of the most widely used Complementary and Alternative Medicine (CAM) therapies used throughout the world. In many countries, HM has a long tradition and the knowledge about local medical plants is ingrained into cultural memory. The WHO estimates that 70–90% of the rural population in developing countries use HM to meet, in part or completely, their health needs [1]. Also, in many developed countries, HM as an element of CAM is highly popular. Therefore, HM is recognized as an essential component of primary healthcare by the WHO [1].

In most developed countries—also in Germany—patients have access to HM via physicians, nonmedical CAM practitioners and on a self-initiated basis. Consequently, diverse healthcare professionals mainly doctors, nurses, pharmacists, and nonmedical CAM practitioners are involved in HM. In Germany, HM is known as one of the five main elements of classic naturopathy (phytotherapy, hydrotherapy, exercise therapy, dietetic therapy, and “life style regulation” therapy) also known as Kneipp therapies. The overall percentage of Germans using HM increased from 52% in 1970 to 70% in 2010 [2]. 

In 2011, about one billion euro were spent on herbal medicine corresponding to approximately 20% of the total expenditure for over the counter (OTC) drugs in Germany. In addition, herbal medicines are sold in drugstores, via internet, and so forth. About 20% of herbal medicine was sold on a prescription-basis and about 80% were sold over the counter [3]. By far the highest sales are made for the indication of respiratory tract infections, followed by cardiovascular disease and gastrointestinal symptoms. However, sales have slightly decreased since the introduction in 2004 of the Statutory Health Insurance Modernization Act, which excluded several groups of drugs including phytotherapeutics from reimbursement. 

German doctors can obtain a postgraduate qualification for “naturopathy,” which includes HM. At the end of the year 2011, 15.949 German doctors had the qualification “naturopathy,” with 70% thereof working in the outpatient sector [4]. However, recent data suggests that far more doctors prescribe or recommend HM to their patients. In a cross-sectional study, more than half of the responding general practitioners (GPs) recommended HM in their day-to-day practice, most of them having no additional qualification for “naturopathy” [5].

Furthermore, HM is often provided by nonmedical CAM practitioners called “Heilpraktiker.” They have to pass an exam on basic medical knowledge to obtain a state license but have no formal training on CAM and HM specifically [6]. While the number of GPs in Germany is decreasing (41.642 GPs in 2011), the number of “Heilpraktiker” has increased over the last years to nearly 32.000 today [4]. Hence, it is clear that Heilpraktikers play a substantial role in Germany for providing patients with HM. 

Altogether these developments suggest that, on the one hand, HM has reached mainstream medicine yet on the other hand, studies show that a substantial percentage of patients do not inform their doctors about their use of HM [7, 8]. Furthermore, international studies suggest that doctors and other health professionals are not well prepared to inform their patients about HM [9, 10]. Considering the interactions between HM and conventional drugs this deficit may affect patient safety and, therefore, should be a point for debate and further research. 

In view of the above, this study is intended to explore the perspectives and experiences of patients using HM in a primary care context. Using a qualitative approach, we focus particular attention on central patient attitudes and motives for using HM, the methods and needs for information and communication and the role of the costs in choices made. 

## 2. Methods

### 2.1. Design of the Study

A qualitative study consisting of guideline-based in-depth interviews was chosen to allow an intensive analysis of patients' perspectives and experiences.

### 2.2. Sample

In 2008, patients were recruited via newspaper advertisements and via a study call on the website of the University Hospital Heidelberg. Inclusion criteria were the use of HM for upper respiratory tract infection in the previous three months, age > 18 yrs and German speaking. We consecutively included all patients meeting the inclusion criteria and with interest to take part in the study. Individual appointments for the interviews were arranged. After 18 interviews, saturation point was reached and recruitment was stopped. 

### 2.3. Data Collection

The in-depth interviews were conducted in the Department of General Practice and Health Service Research, University Hospital of Heidelberg, Germany. The interviews were semistructured based on a predefined interview guideline and conducted by a doctoral research student. Each interview lasted between 15 and 45 minutes. All interviews were recorded digitally and transcribed verbatim.

The results presented in this article are based on the following questions from our interview guideline.

- How did you come to use Herbal Medicine?
- What were your expectations towards Herbal Medicine?
- What are your main sources of information about Herbal Medicine?
- What role does the cost of Herbal Medicine play regarding your decision to use it?
- Do you tell your doctor that you use Herbal Medicine?

The aims of the study were explained to each interviewee. The interviewer ensured that each aspect of the questions was explained sufficiently, so that no questions or misunderstandings remained. 

### 2.4. Ethics Approval

The study was approved by the ethics committee of the Heidelberg Medical Faculty (approval number 394/2006).

### 2.5. Data Analysis

The interviews were carried out in 2008. Analysis was conducted using the software ATLAS.ti. Key issues were identified, summarized, labeled with codes and sorted into main and subcategories based on the qualitative content analysis technique from Mayring [11]. The aspects of interpretation and categories were developed based on the material. For each category, a typical quotation was selected. The interviews and analyses were conducted simultaneously, so that researchers could control for topic saturation. Disagreements during coding process were discussed until a consensus was reached. The quotations cited here were translated into English by Pia Weiss and cross-checked by SJ and BM. 

## 3. Results

Eighteen patients participated in the study. The sample of the 18 interviewees is shown in Table 1. Among the interviewees 16 were female and two were men. Mean age was 46.5 years, nearly half of the participants were employed, four were students, and five were retired. The most common way of recruitment was via newspaper advertisement, followed by the internet and word of mouth.

The categorical framework developed in the course of the qualitative content analysis is displayed in Tables 2, 3, and 4. In the following, all categories are explained and examples of typical quotations are given for the subcategories.

### 3.1. Attitudes, Values, and Motives

Underlying attitudes, motives, and values for using HM were categorized in five main groups: “medical culture,” “personal experience,” “subjective theory of disease,” “defensive attitude towards conventional medicine,” and in “active role” (Table 2). 

#### 3.1.1. Medical Culture

Among the main category “medical culture” comments regarding patients' desire to maintain traditional knowledge can be found. The fact that herbal remedies have existed for hundreds of years is perceived as proof of evidence by some patients. One patient describes it as an “*implicit knowledge of society.” *

* “Those are ancient natural remedies which are regaining their popularity since there's simply an abundance of those chemical things, all those chemical pills.” *

* “They don't meddle around with your system quite like chemical drugs and knowledge about them has been around for a long time. Also, about that […] society knows if they are of good use or not, doesn't it?” *

Some of the patients are reporting that their mothers or grandmothers have already been using herbal therapies and that it is “family tradition.”

* “Well, my mother didn't send us to the doctor right away then or fed us antibiotics, rather she tried herself, you know, with home remedies.” *

Not only family but also the social environment as a mediator of “medical culture” may play an important role in motivating patients to use herbals. One patient uses the expression “trend” in this context. For others, the use of herbals is an indicator of “higher education.”

* “Maybe if you studied it and lived in an environment where everyone has an idea, that this symptom could be remedied with this tea, and so forth, then it simply amplifies, I think, over the course of time.”*

* “If I knew that only uneducated people or readers of the yellow press believed in natural healing then I wouldn't take it as seriously, too.” *

Moreover, doctors as main representatives of “medical culture” play a central role in influencing patients' attitudes towards herbal medicine.

* “It does depend on my doctor. I just have trust in him now […] if he would say we're trying that now […] then I would take it and try it.” *

A lot of patients using herbals refuse to have exaggerated faith in medical and scientific progress.

* “I have been reading a lot of articles lately that were critical towards the current practice in medicine […] there's such an overstated belief in progress. And if people resort to herbal medicine they say “We don't even want modern medicine, we want the time-tested.””*

#### 3.1.2. Personal Experience

In general, interviewed patients expressed expectations based on their previous experiences regarding HM. Patients commented on their experiences with HM primarily in comparison with conventional drugs. Experiences are described as “better and with a more lasting effect,” “a more gentle effect,” “fewer side effects,” “sustained, not only symptomatic cure.”

* “I think their effects are gentler, that they are - that they don't have as many side effects, that you may get a longer-lasting effect if you are taking them on a regular basis.” *

There were many comments on preventive effects of herbals. However, some patients bring into consideration that it is difficult to say whether the effect is caused by herbals or not.

* “And as soon as I get that feeling, like I am outside for more than an hour, I prepare a cup of tea for myself right away when I get home, as a preventive measure.” *

* “Of course you can't prove it afterwards. As in I did not get a cold because I took that. And then if you get one you say ‘Now I got sick anyway'.” *

For some patients herbals are always the first course of action if feeling symptoms.

* “Well, as first course of action I almost always prefer natural remedies. Especially for the gastro- intestinal tract, but also for cold-like illnesses, be it a head cold, a cough, hoarseness, a sore throat.”*

Some patients mention that there is more time needed with HM in comparison to conventional medicine. Many patients report a longer duration of treatment but a continuing effect of herbals. A few of them think that this could also be an advantage because patients have to cure themselves properly.

* “And with herbs and teas and all those alternative treatments it takes longer for it to go away sometimes but then it really is gone.” *

* “If I have the time for it and really recover from it then I do drink tea all day long or I try to stay warm, and well, keep to the rules that are known to help recovery.” *

Some interviewees explicitly explain that they use HM as a substitute for antibiotics in the case of respiratory tract infections.

* “Well to me it's about the question antiobiotics [sic] or no antibiotics. And that's actually my motivation there to get around the antibiotics […] well, since I have been taking that early on I have, if I remember correctly, not taken any more antiobiotics [sic]. And before, I did that on a regular basis.” *

Some patients describe that herbals appeal to the senses and evocate positive memories.

* “Well what is pleasant is that it does have an immediate effect as you have a taste of eucalyptus [sic] in your whole mouth, well in your nose, in your mouth. So you instantly feel that it's working." *

* “You do already feel better with a cup of tea in your hands. And it heats you up from the inside for sure. Though I don't know if it really does that. Yet, with a cold I don't really know if the tea really plays such a big part. Maybe it has a soothing effect on your throat or something like that, or it's simply relaxing." *

#### 3.1.3. Subjective Theory of Disease

Most of the interviewed patients prefer a more “natural way of healing” embedded in a holistic approach. This opinion was often combined with the idea that disease is caused by an imbalance of the organism and HM would act as an aid for self-help, for example, strengthen the body's natural defense system. In this sense herbals provide a causal way of healing.

* “So you don't just fight an illness, but treat the whole person as such. I have studied the topic quite thoroughly.” *

* “So they support the body's abilities but not interfere too heavily so that they support the body's healing power” *

HM as therapy which does not disturb the body's balance is seen as one of the main differences in comparison to conventional medicine.

* “When the body gets out of balance I help it get back to its balance rather than I just take something and not restore its balance but fight a symptom instead.” *

#### 3.1.4. Defensive Attitude towards Conventional Medicine

Some of the interviewed patients have developed a defensive attitude toward conventional medicine, which is mainly based on side effects they have experienced taking antibiotics or other conventional drugs.

* “Sure, everything you take regularily [sic] has side effects. I think, however, that you can downright poison yourself with conventional medicine.” *

* “I was prescribed so many antibiotics that I developed an allergic reaction to penicillin [sic]. Now, those agents are extremely harmful, actually, I am left with an infection of the intestines every time.” *

Further, the lack of time of conventional doctors is given as an explanation for the preference of HM. A few patients report that they do not feel taken seriously by their conventional doctors.

* “And one of the reasons is doctors not taking enough time, for sure.” *

One patient expressed a great mistrust towards pharmaceutical companies as an explanation for his defensive attitude towards conventional medicine.

* “For example, I made the decision not to go to any doctor meeting with medical representatives. And every doctor meets them. So you have to go to an alternative practitioner separately, though even they meet them […] I would rather try charmstone therapy than have a medical representative tell me what drugs I to take because that's not objective information […] what would be important to me were to improve the doctor-patient relationship, that for example there would be a law that they cannot advertise at doctors' offices.” *

#### 3.1.5. Active Role

In many interviews an elevated health awareness was expressed by the interviewees.

* “No, you have to have a little patience, but I do take it from the start. I don't even let it get that far and then it's taken care of very soon too. You just have to have a little feel for it.” *

An important reason stated by some patients to use HM was to take over an active role in the process of treatment combined with a feeling of high autonomy and self-responsibility. A few patients see HM as a possibility to reduce consultations with a doctor.

* “Sometimes it doesn't work any other way, but essentially, you can take many matters into your own hands. Maybe that's one of those instances where you have to think for yourself and not have yourself get back on your feet with meds.” *

* “And I think I am the same as many others, that I decide to just treat myself, if that harms me I will bear responsibility for it and won't hand it over.” *

Additionally, feelings of curiosity, inquisitiveness, fascination, or just the search for something else were reported by some of the patients as reasons to use HM.

* “When I hear of a plant that's new to me. “Stevia” I heard of for the first time recently. I look it up after. And find out if someone has done any research about it or what effects it has. If I do find someone who knows something about it then I interrogate him thoroughly.” *

### 3.2. Communication and Information

Statements on communication and information were categorized in four categories “doctors' approach,” “quality of communication,” “relevant elements of information,” and “sources of information” (Table 3). 

#### 3.2.1. Doctors' Approach

According to the patients' experiences doctors' attitudes concerning HM range from categorical refusal, dogmatism, unconcern to sincerity, and a positive conviction.

* “My doctor at home was fairly conservative.” *

* “Yes, he does know about that [that I am taking herbal medicine]. I think he doesn't care about it, pretty much.” *

* “My current GP does have a good attitude but he also has further training in herbal medicine. But I am very happy now there is a response.” *

One patient expresses a deep mistrust towards conventional doctors because he thinks that they are henchmen of the pharmaceutical industry.

* “I think of doctors as the henchmen of pharmaceutical companies, too. Politicians rather like to gather their belongings. They also profit from it.” *

#### 3.2.2. Quality of Communication

Some patients report their doctors being very open or even enthusiastic towards trying out new approaches to treatment with HM. They feel accepted by their doctors in the sense of a shared decision.

* “He is always thrilled whenever I get him something like that [information about HM].” *

* “I know from acquaintaces [sic] who can afford a real homeopath, partly they don't go there because of the medicine, but because he is taking his time and there's a real conversation, you can talk to him about everything, and your problems and fears can be treated with drugs, too. It just felt better somehow…” *

Other doctors seem to have a rather patriarchal style communicating with their patients about HM.

* “I used to have a different doctor too: ‘I am the doctor, you can't tell me what to do.' And so on.” *

Some patients describe a miscommunication between diverse providers (doctor, CAM practitioners, pharmacists, and pharma companies) and between doctors and patients.

* “I've noticed many doctors being very self-opinionated. They will say ‘I am prescribing this to you and you don't have a say in it'. And when I inquire ‘But what kind of side effects does this have, which alternatives are there?'. It's like that […] they don't like that at all, to be questioned.” *

Placebo and nocebo-effects (especially induction of fear) play an important role in communication about HM.

* “I can't say I have certain expectations towards a specific drug. Additionally, I can imagine that many herbal drugs are simply ineffective or just have a placebo-effect. Well actually […] I do have expectations. When I am taking something like that I always have a sense of having taking something without resorting to a chemical cocktail and I also think that's the reason why many others do that too.” *

* “…But then you have a look on the patient information leaflet and see how many side effects there are.” *

#### 3.2.3. Sources of Information

The sources of information of our interviewed patients were widespread across all media and healthcare professionals, family, friends and colleagues, advertising as well as patient information leaflets.

* “Yes, I sometimes read what's in the paper or in magazines, or I watch TV and listen to what my mother says, or my grandmother, what kind of experiences they've had in the family circle. […] basically, that's where I get my information from” *

* “Ok sure, but if I asked about it I would ask a doctor. Or whenever I have a colleague who knows about that stuff […] I have the internet and I read medical journals, of course.” *

* “Well I have to say those magazines in pharmacies are pretty valuable nowadays.” *

* “While at the pharmacy and with friends, you just talk about that, because you […] I am a scientist and work with quite a lot of doctors, so mostly acquaintances or apothecaries.”*

* “Well, I get it from the patient information leaflet or afterwards, during the talk with the doctor.” *

* “I was in a self-help group. I was there when there was a meeting for people affected by fibromyalgia or rheumatism somewhere […]” *

* “I am a member of a society for alternative medicine. And if I don't know something myself then I will ask the alternative practitioner.” *

#### 3.2.4. Relevant Elements of Information

Some patients wish to have more information about indications, side effects and mechanism of action of some herbal preparations. They would like to have specific recommendations about dosage with regard to side effects and specific conditions such as pregnancy.

* “You don't find a lot about herbals in the patient information leaflet. They always write ‘no side effects' or ‘allergy' and that was it.” *

* “It is pretty difficult with herbal medicine because there is so little about the side effects in the patient information leaflet. Sometimes, I can't imagine there being so little side effects if it's an herbal drug.” *

* “I do know that [name of a herbal preparation] is highly problematic for pregnant women because there is some kind of active ingredient in there which is just problematic. I just think in this respect I would not think of this as any safer than the meds of conventional medicine.” *

Furthermore, uncertainty exists about possible quality differences in drugs and possible consequences regarding side effects.

* “I still prefer them [plants] from the pharmacy, however, because they have certain quality standards they have to adhere to. The prepackaged thyme tea, for example, is sometimes offered in the drugstore but I have made the experience that it wasn't so good.” *

Patients want safe herbals with a lack of toxic remnants.

* “That they're pure, that they have a certain quality, are free of pesticides and other harmful substances […] basically what you would expect of other drugs, too.” *

### 3.3. Cost

Patients' comments on costs were categorized in two main categories: “cost for the individual” and “cost for the public” (Table 3). 

#### 3.3.1. Cost for the Individual

Most patients seem to be willing to pay extra for HM, because health is worth the money, although some admitted that they would only pay for proven therapies. A few patients complained that prices for some herbals are far too high.

* “With things like health or well-being, I don't care. If I know it will help me I will gladly spend the money.” *

* “I think it's okay for it to be more expensive than regular painkillers because I think that it has a higher value. But it can't cost like double. Under ten euro is manageable.” *

* “For example with gingko […] I had been interested in it, for studying it's quite good. But it was too expensive […] and I think that it is a kind of trend […] it simply was too pricey for me.” *

A few patients are looking for alternative ways to get herbals. One patient said that he orders herbals on the internet because it is less expensive. Another patient collects plants in the surroundings and cultivates medicinal plants in his own garden.

* “The pharmacy is too expensive. I found a way of getting it directly from the factory.” *

* “Getting the herbs yourself, I did that too, but it's a bit more difficult and you have to be in the right area. In Heidelberg, it's somewhat difficult, but generally, I get them from the pharmacy.” *

* “And like I said, one always has a shrub of peppermint tea in one's garden.” *

#### 3.3.2. Cost for the Public

Some of the patients expressed their anger about the lack of reimbursement of HM although herbals are less expensive compared to most of the conventional drugs. They argue that access to HM should not be a question of cost.

* “When they were saying you had to pay for your own medicine I was definitely upset because I thought it costs so little compared to the other drugs that are being prescribed. It's a shame that it's not being paid for anymore […]” *

* “I would do that [visit the alternative practitioner] if I could afford it […] policy is always aiming at the patient paying for these things himself […] but it's just a question of cost […] I don't have a lot of money and healthy food is important, too. I mean I can't spend money on the alternative practitioner if I don't have money to pay for my food.” *

In the patients' opinion HM is a cost efficient therapy.

* “I can say something about the healthcare system. What I find a bit odd is that this herbal drug and the homeopathic one for your throat was recommended to me by an ENT specialist … and since I have been taking it I have neither had tonsilitis nor bronchitis, which I had had regularily [sic] before and that was with antiobiotics [sic] and all that crap. I got sick right after. That ENT specialist hasn't seen me since and she doesn't make any money, since she has advised me so well, she doesn't make any money off me, because I am done […] And that I find considerable. Because there's an error in the system somehow.” *

Patients express in their own words general problems of modern health systems based on competition and profit maximization leading to oversupply, undersupply, and inappropriate healthcare.

* “Truthfully, I don't find it acceptable for them to be allowed to influence doctor so much because health isn't a product that can be advertised for like for a car or something, if you know what I mean. And I think that it's not only me, but also the other patients that are becoming pretty suspicious and are just looking for alternatives. Well, that's simply something that is so noticeable and negatively so. And I think to myself, that's why phytotherapy is booming.” *

## 4. Discussion

This study reveals some new and specific aspects for HM from the patients' perspective which may supplement the results of preceding studies. The interviewed patients feel a traditional link to HM nourished by experiences in the family, by friends or persons in their social environment. A major part of the patients highlight the active role and the feeling of more autonomy using HM fitting well with a higher health awareness, curiosity, and motivation to try out something else. Most interviewees have the experience that HM has softer and slower effects, but in a more causal and, therefore, more sustainable way. HM is regarded to have fewer side effects compared to conventional medicine. However, some of the interviewed patients do not feel sufficiently and reliably informed about indications of HM, dosage and possible side effects, or interactions with conventional drugs. Furthermore, our results show that patients draw their information from multiple sources: the media, family and friends, healthcare professionals including health insurances and self-help groups. Patients' experiences with doctors regarding HM are varied, ranging from active recommendations of specific preparations to categorical refusal of HM. Patients' opinions regarding costs all point in the same direction: a lack of understanding that HM is not being reimbursed by health insurances, but a readiness to pay extra for HM.

Our results are in good agreement with preceding qualitative studies exploring patients' views on HM, revealing autonomy and patient-practitioner collaboration as main themes in this context [12–14]. In a UK interview study with 19 adults regularly consulting medical herbalists participants reported that, in comparison to conventional medicine, HM satisfied their expectations of healthcare because it has greater consistency with their own understanding about health, illness, and healthcare [12, 13]. Also, in our study the “subjective theory of disease” was identified as a main category, being of great importance for patients. Another qualitative study from the UK with female patients demonstrates severe information deficits regarding herb-drug interactions [14]. From our study it can be demonstrated that patients draw on a number of information sources with the risk of wrong or missing information. Partly they are complaining about their doctors' lack of information about HM. A lack of knowledge may be also suggested considering results of international studies. According to a survey among GPs in the United States the overwhelming majority (84%) thought they needed to learn more about CAM to adequately address patient concerns [9]. A cross-sectional study among doctors, pharmacists, nurses, and dietitians has shown that physicians assess their levels of knowledge about HM as moderate and their communication skills as poor [10]. A systematic review about HM and pharmacists points in the same direction. Pharmacists do not perceive their knowledge about herbals to be adequate [15]. Comparable studies for Germany have not yet been conducted.

In some countries attempts have been made to overcome this problem with educational programs. Fact sheets about herbals were developed and tested among 92 GPs within a research project in Australia. The results of this study show that fact sheets increased knowledge and improved communication with their patients about those specific herbals [16]. In Canada, core competencies in the education of pharmacists about HM have been developed and agreed among pharmacy educators [17]. In a US study an E-curriculum about herbs led to significant and sustained improvements in clinicians' expertise about herbs regardless of the delivering strategy [18]. Another way could be the use of the internet as a scientifically qualified source of information about HM for patients. In Germany, first projects have already put into practice, for example, www.phytodoc.de. However, the impact of those platforms needs to be assessed in the future.

Educating health professionals about HM should include the concept of “meaning effects” [19]. According to our main categories “medical culture,” “subjective theory of disease,” and “active role” HM is more than just taking a pill. Also, clinical studies point in the same direction and show that—similar to other CAM therapies—“meaning effects” (or so-called placebo effects) play a major role in the therapeutic success of HM. A recently published study investigating placebo effects in patients with common cold receiving placebo or *Echinacea* supports the general idea, that beliefs and feelings about treatments may be important and should be taken into consideration when making medical decisions [20]. The findings from our qualitative study may provide a deeper understanding of this idea. For example, some comments within our interviews show that herbals appeal to the senses and evocate positive memories—a potentially potent way of encouraging the healing process and promoting health.

Furthermore, our findings show that the social environment in this context is highly important. This is also supported by the findings of an Australian study among older women showing that CAM use was reflective of the personal beliefs of the women and members of their close social networks. The differences between women living in rural versus urban regions observed in this study have been attributed to the difficulties inherent in accessing certain types of CAM in rural areas [21].

According to the WHO medicines Strategy 2008–2013, the integration of HM into the national health systems should be facilitated with a focus on better regulation of products and providers [22]. In Germany, the time has come to educate health professionals working in primary care about the basics of HM. Special emphasis should be placed on frequent indications such as respiratory tract infections and conditions where herb-drug interactions are probable, such as depression. It has to be ensured that information provided by the different professionals is consistent and in line with evidence—as far as evidence exists. This process should be promoted by universities in cooperation with the professional bodies of health professions in order to maintain independence of the pharmaceutical industry. Ideally, learning objectives regarding HM should be integrated into residency training curricula of GPs to ensure each GP having a basic knowledge in HM. A challenge for doctors will be to understand the individual needs of each patient regarding HM. This may include clarification of and communication about possible side effects and interactions of herbal drugs. This is particularly important in multimorbid patients with multimedication, furthermore, in patients with specific indications and while supporting them in their active role and self-management. All that is only possible if reimbursement of herbal medicine by the statutory health insurance will be reintroduced. Indeed, there is some evidence by health economic studies making this an interesting option also for health policy. As indicated by a Dutch study, patients whose GP had additional CAM training generate up to 30% less healthcare costs [23]. This may be caused by the more active role patients have in CAM. As our results show, HM can increase self-responsibility of patients and, hence, doctor visits may become less frequent. Furthermore, HM may reduce overuse and misuse of antibiotics, for example, in treatment of viral respiratory tract infection and therefore prevent negative consequences of antibiotics' overuse. However, this should be subject of future studies.

Our study may have some limitations because we mainly interviewed female patients having had positive experiences with HM. However, our aim was not to draw conclusions on a representative basis but to explore the experiences of patients with HM and to understand their motives for using HM. 

## 5. Conclusion

The use of HM meets patients' personal understanding about health and illness and their need for autonomy and self-care. However, there is a demand for better and consistent information on HM. The main challenges in the context of primary healthcare are to promote and upgrade the knowledge and skills of the providers about HM to ensure patient safety and to support patients in their self-management. Appropriate strategies for education have to be developed and tailored to the specific needs of health professional groups. At the same time, further research under inclusion of patients' views is much-needed in order to improve the evidence base for HM. 

## Figures and Tables

**Table 1:** Study sample of participating patients (*n* = 18).

Gender	
Female	16
Male	2

Age	
Mean	46.5 years
Range	Min. 23/max. 82 years

Occupation	
Employed	8
Retired	5
Students	4
Housewife	1

Way of recruitment	
Newspaper	8
Internet	6
Word of mouth	4

**Table 2:** Attitudes, values and motives (main and subcategories).

Main category	Subcategory
(A) Attitudes, values and motives	

(A1) Medical culture	*∙* Maintenance of traditional knowledge
	*∙* Long experience as proof of evidence
	*∙* Family tradition
	*∙* Social trend
	*∙* Marker of higher education
	*∙* Doctors as main representatives
	*∙* Refusing exaggerated faith in progress

(A2) Personal experience (mainly expressed in comparison to conventional medicine)	*∙* Better effects
	*∙* More gentle effects
	*∙* Fewer side effects
	*∙* Sustained, not only symptomatic cure and
	*∙* Slower onset of action/more time with herbals
	*∙* Preventive effects
	*∙* Herbals as primary treatment approach
	*∙* Substitution of antibiotics
	*∙* Herbals appeal to the senses/evocate positive memories

(A3) Subjective theory of disease	*∙* Holistic approach
	*∙* Herbals strengthen body's natural defence system
	*∙* Herbals do not disturb the body's balance
	*∙* Causal way of healing

(A4) Defensive attitude towards conventional medicine	*∙* High risk of side effects (especially antibiotics)
	*∙* Conventional doctors' lack of time
	*∙* Patients do not feel taken seriously
	*∙* Mistrust towards pharmaceutical companies

(A5) Active role	*∙* Elevated health awareness
	*∙* Active role
	*∙* More autonomy
	*∙* High self-responsibility
	*∙* Curiosity/inquisitiveness
	*∙* Looking for other sources of help

**Table 3:** Communication and Information (main and subcategories).

Main category	Subcategory
(B) Communication and information	

(B1) Doctors' approach	*∙* Categorical refusal
	*∙* Dogmatism
	*∙* Unconcern
	*∙* Sincerity
	*∙* Positive conviction
	*∙* Henchmen of pharmaceutical industry

(B2) Quality of communication	*∙* Feeling accepted by their doctors
	*∙* Shared decision-making
	*∙* Patriarchal style
	*∙* Miscommunication
	*∙* Placebo-effects
	*∙* Nocebo-effects (e.g., induction of fear)

(B3) Sources of information	*∙* Family/social environment
	*∙* Doctors
	*∙* Pharmacists
	*∙* CAM practitioner
	*∙* Media (TV, newspapers, internet, brochures)
	*∙* Literature (books, journals)
	*∙* Advertising (pharmacy brochures)
	*∙* Patient information leaflets

(B4) Relevant elements of information	*∙* Indication
	*∙* Side effects
	*∙* Mechanism of action
	*∙* Dosage recommendations
	*∙* Safety recommendations for pregnancy
	*∙* Quality differences in drugs and possible consequences regarding side effects.
	*∙* Declaration of toxic remnants

**Table 4:** Cost (main and subcategories).

Main category	Subcategory
(C) Cost	

(C1) Individual cost	*∙* Health worth the money
	*∙* Additional cost only for evidence-based therapies
	*∙* High cost of some herbals
	*∙* Difference in cost
	*∙* Internet order
	*∙* Collecting plants in regional environment
	*∙* Home-cultivated plants

(C2) Public cost	*∙* Lack of reimbursement,
	*∙* Herbals less expensive than conventional drugs
	*∙* Access to herbals as a question of cost
	*∙* Cost efficiency of HM,
	*∙* Reduced frequency of doctor visits
	*∙* Oversupply, undersupply, inappropriate healthcare
